# Role of the single nucleotide polymorphism rs7903146 of *TCF7L2* in inducing nonsense-mediated decay

**DOI:** 10.1186/2193-1801-3-41

**Published:** 2014-01-22

**Authors:** Nathalie Nicod, Marta Pradas-Juni, Ramon Gomis

**Affiliations:** Diabetes and Obesity Research Laboratory, Hospital Clinic, University of Barcelona, IDIBAPS, Rosselló 153, Barcelona, Spain

**Keywords:** TCF7L2, SNP rs7903146, Type 2 diabetes, Alternative splicing, Nonsense-mediated decay, Islets, Beta-cell

## Abstract

**Background:**

The single nucleotide polymorphism (SNP) rs7903146 (C/T), located in intron 4 of the transcription factor 7-like 2 gene (*TCF7L2*), has been associated with an increased risk of developing Type 2 Diabetes, although the molecular mechanism remain elusive. The *TCF7L2* gene is alternatively spliced but an association between genotype and splice variants has not been shown convincingly. We hypothesized that a yet unknown extra exon, containing either the C or T genotype of the SNP rs7903146, could introduce a premature stop codon and consequently result in nonsense-mediated decay (NMD).

**Findings:**

Running the sequences C and T of the SNP region in different servers we found that the two alleles could display differential recognition by splicing factors. The C variant showed the possible inclusion of an unknown exon. This unknown exon contained a stop codon and thus could induce NMD. We then determined that the splicing pattern in isolated mouse islets and MIN6 cells was similar to that in human pancreatic islets. Therefore, we used MIN6 cells to study the splicing of human intron 4: two mini-genes of intron 4 containing either the C/C genotype or the T/T genotype were transfected into MIN6 cells. Our constructs were spliced normally, excluding intron 4, but we did not observe the presence of an extra exon with either construct.

**Conclusions:**

We found that an extra exon could theoretically exist, although we were not able to capture it in our model. A better model is needed to determine whether a theoretical extra exon can induce NMD.

## Introduction

Grant et al. previously reported that the single nucleotide polymorphism (SNP) rs7903146, consisting of a C to T nucleotide change in intron 4 of the transcription factor 7-like 2 gene (*TCF7L2*), was strongly associated with the risk of developing type 2 diabetes (T2D) in Icelandic, Danish, and USA populations (Grant et al., 2006). Many other studies have confirmed these findings in other populations (Helgason et al., 2007; Florez et al., 2006; Lyssenko et al., 2007; Dahlgren et al., 2007), but the mechanisms remain largely unknown. *TCF7L2* and *insulin* mRNA expression in human pancreatic islets was increased in individuals carrying the at-risk T allele (Lyssenko et al., 2007), however glucose-stimulated insulin secretion was inversely associated, suggesting that TCF7L2 protein might influence post-transcriptional events of insulin expression. Individuals with the T allele had an increased proinsulin to insulin ratio (Loos et al., 2007; Stolerman et al., 2009). Proprotein Convertase 1 (PC1) is partly responsible of this ratio since it cleaves proinsulin to obtain mature insulin in the beta-cell. Therefore, these results suggest that the SNP might modulate proinsulin processing in beta-cells by modulating PC1 expression. Indeed, the promoter of PC1 was shown to have theoretical TCF-binding sites (Loos et al., 2007; Stolerman et al., 2009), thus TCF7L2 protein could modulate insulin processing, indirectly through modulating PC1 expression. In vivo, the T allele predicted hyperglycaemia which was associated to reduced insulin secretion but not to reduced insulin sensitivity (Cauchi et al., 2006; Wang et al., 2007), indicating that indeed the SNP affects beta-cells and insulin production/secretion but does not affect insulin resistance of peripheral tissues. The latter studies did not look at plasma proinsulin levels, which probably would have been increased, and which would have indicated that it is the conversion of proinsulin to insulin that is affected in individuals with the T allele.

*TCF7L2* is encoded by 17 exons, five of which are alternatively spliced (exons 4, 13, 14, 15, and 16) and show tissue-specific expression in both humans (Mondal et al., 2010) and mice (Weise et al., 2010). Since the SNP rs7903146 is found in a non-coding region and several unknown protein factors bind to the C allele but not the T (Cauchi et al., 2008), we hypothesized that different splicing factors could bind to the T allele, relative to the C allele, and alter the splicing pattern of *TCF7L2* mRNA. Indeed, transcripts retaining exons 14 and 15 were significantly correlated with rs7903146 (Prokunina-Olsson et al., 2009; Mondal et al., 2010), however it is not clear how a SNP in intron 4 could affect splicing of exons 14 and 15. We hypothesized that a yet unknown exon with a premature stop codon could be alternatively spliced in beta-cells with the T genotype. A premature stop codon in one of the genotypes could induce nonsense-mediated decay (NMD), and thus result in different expression of total *TCF7L2* mRNA in T carriers, as seen in several studies (Pang et al., 2011; Lyssenko et al., 2007). This could explain altered proinsulin processing, since the promoter of PC1 has theoretical TCF-binding sites (Loos et al., 2007).

## Materials and methods

Isolation of mouse islets and culture of MIN6 cells was carried out as previously described (Casas et al., 2008). MIN6 cells were transfected with lipofectamine (Invitrogen).

The study was approved by the Ethics Committee of Clinical Research of the Hospital Clinic, Barcelona.

### Splicing analysis

RNA from both, MIN6 cells and isolated mouse islets, was extracted with the NucleoSpin® RNA kit (Macherey-Nagel) and cDNA was synthesized with Superscript® III Reverse Transcriptase (Invitrogen) according to manufacturer’s instructions. Alternative splicing was analyzed by PCR: exon 4 splicing was determined using primers designed for exons 3 and 5. Similarly, alternative splicing at the 3′ end was determined with primers for exons 12 and 17. PCR products were run on an agarose gel, bands were excised and cleaned with NucleoSpin® Gel and PCR Clean-up kit (Macherey-Nagel). Bands were sequenced with the BigDye® Terminator v3.1 Cycle Sequencing kit (Applied Biosystems).

### Constructs

**Figure 1:**
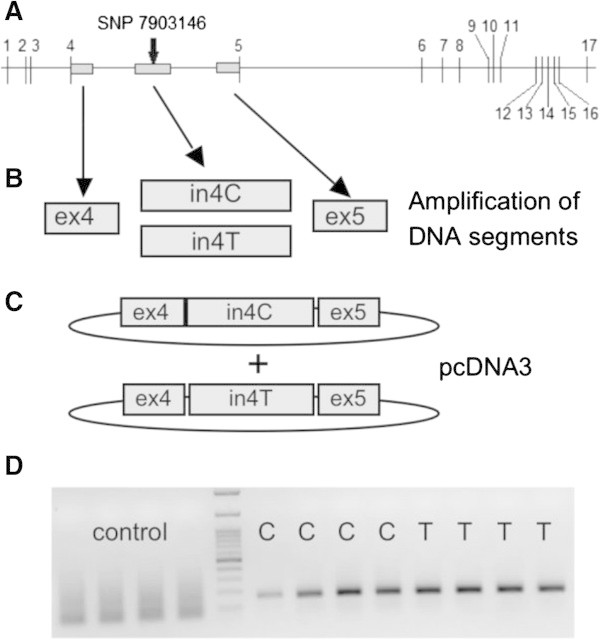
**Alternative splicing of intron 4 mini-gene containing the C/C or T/T genotype. A**: human *TCF7L2* gene, exons are represented by vertical lines. Grey boxes represent the parts of the gene that have been cloned to create the mini-gene **B**: amplification of four DNA segments (ex4: segment containing exon 4 and part of the beginning of intron 4; in4C: part of intron 4 containing the SNP with the C/C genotype; in4T: part of intron 4 containing the SNP with the T/T genotype; ex5: segment containing the end of intron 4 and exon 5). **C**: Two pcDNA3 plasmids were obtained with either the C/C (ex4-in4C-ex5) or the T/T (ex4-in4T-ex5) genotype. **D**: 72 h after MIN6 cells were transfected with plasmids, RT-PCR and an agarose gel were run, n = 4 independent experiments.

DNA was amplified with Platinum® *Taq* DNA Polymerase (Invitrogen) to make four segments of DNA. ex4: segment containing exon 4 and part of the beginning of intron 4; in4C: part of intron 4 containing the SNP with the C/C genotype; in4T: part of intron 4 containing the SNP with the T/T genotype; ex5: segment containing the end of intron 4 and exon 5 (Figure 1, primers available upon request). The in4C and in4T constructs were sequenced and differed only in the nucleotide intended. All four constructs (ex4, in4C, in4T, ex5) were each ligated with a pGEM®-T Vector System (Promega), transfected and grown in Subcloning Efficiency™ DH5α™ Competent Cells (Invitrogen). Clones were digested, cleaned, ligated, transfected, and grown again in Competent Cells. Plasmids containing ex4-in4C-ex5 or ex4-in4T-ex5 were obtained and the pGEM®-T vector was replaced by a pcDNA3 vector.

## Results

### Characterization of alternative splicing in mouse islets and MIN6 cells

**Figure 2:**
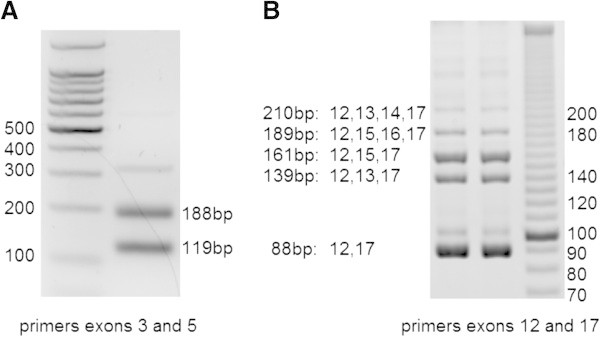
**Alternative splicing of** ***Tcf7l2*** **mRNA in isolated mouse islets.** Agarose gel from RT-PCR with primers in exons 3 and 5 **(A)**, and primers in exons 12 and 17 **(B)**. **B**: both lanes are from a similar PCR product (two bands were necessary to obtain enough product for the low intensity bands). Exon sequences and base pair lengths are shown for all the bands that were sequenced.

Alternative splicing of *Tcf7l2* was similar in isolated mouse islets (Figure 2) and MIN6 cells (results not shown). We determined that exons 4, 13, 14, 15 and 16 were alternatively spliced. PCR of exon 4 revealed two bands, which after purification and sequencing, corresponded to the inclusion (188 bp) and exclusion (119 bp) of exon 4 (Figure 2A). Similarly, alternative splicing in the 3′ region was examined using primers in exons 12 and 17 and five bands were isolated. Three major bands corresponded to 12–17 (exon 12 spliced into exon 17) (88 bp), 12-15-17 (161 bp), and 12-13-17 (139 bp) and two minor bands corresponded to 12-15-16-17 (189 bp) and 12-13-14-17 (210 bp) (Figure 2B). In humans islets, Mondal et al. showed that the major variant was 12–17, and the other variants present were 12-13-17, 12-15-17, 12-15-16-17 (Mondal et al., 2010). Moreover, we found the same deletion of 12 nucleotides in exon 7 and an insertion in exon 9, previously described in other tissues (Duval et al., 2000). Therefore, our results show that the splicing pattern in isolated mouse islets and MIN6 cells is similar to that in human islets, suggesting that MIN6 cells are a good model in which to investigate alternative splicing of a mini-gene containing human intron 4.

### Theoretical determination of an extra exon inducing NMD

**Figure 3:**
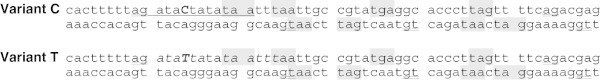
**Sequence of part of intron 4 around the C/T SNP rs7903146.** Both genotypes the C and the T variant are shown: the C and T nucleotides of the SNP are shown in capital and bold. Possible stop codons which if inserted could mediate NMD are in grey, possible 5′ (ag) and 3′ (gt) splice sites for the hypothesized extra exon are double underlined (possible exon includes nucleotides from ag to gt), possible ESE are underlined in the C variant and possible ESS are in *italic* in the T variant.

Splicing factors bind to exon splicing enhancer (ESE) motifs to include an exon, or to exon splicing silencer (ESS) motifs to exclude an exon. Running the two variant sequences of rs7903146 in the regulatory motif search server developed by Gil Ast (http://astlab.tau.ac.il) and a server developed in Roderic Guigó’s group, we found that the two variants could display differential recognition by splicing factors. The C variant could promote splicing, due to the presence of an ESE motif, and thus cause exonisation of a sequence creating a new exon inside intron 4, (Figure 3 variant C, ESE is underlined). This could lead to an mRNA containing a premature stop codon (grey shadow in Figure 3), which could possibly undergo NMD and lead to reduced levels of mRNA. The T variant on the other hand displays motifs that are not present in the C variant which correlate with splicing repressive sequences when present in exons, ESS (Figure 3 italics in variant T). Thus, we hypothesized that the polymorphism rs7903146 could generate the exclusion of a yet unknown small alternative exon, Figure 3. This alternative exon would contain a stop codon, which would then cause NMD of the short mRNAs produced. Indeed, NMD, which is the rapid degradation of short mRNAs, is difficult to observe because short mRNAs are degraded rapidly before it is possible to extract them from cells for analysis (McGlincy and Smith, 2008). Thus the C variant contains ESE sequences and the T variant contains ESS sequences, suggesting a possible extra exon in the C variant and the skipping of this exon in the T variant. We undertook to investigate the existence of this extra exon.

### Alternative splicing of intron 4 mini-genes

In order to study the possibility of an extra exon in intron 4 we needed to clone intron 4. Since human intron 4 is very long (80 kbp) and difficult to clone, we created a shorter version of human intron 4 which included three different segments of this intron: one 581 bp segment contained exon 4 and the beginning of intron 4 (ex4), then a 1000 bp segment consisted of intron 4 including the C/C or T/T genotype in the middle (in4C or in4T), and finally a 500 bp segment contained the end of intron 4 and exon 5 (ex5) (Figure 1, grey boxes). We obtained two constructs with either the C/C (ex4-in4C-ex5) or the T/T (ex4-in4T-ex5) genotype (Figure 1C). The two constructs were each inserted in a pcDNA3 Vector System and transfected in MIN6 cells (an empty pcDNA3 Vector was used as control). The transfection of these two constructs (C and T) in MIN6 cells should undergo similar splicing as in humans, since we found that *TCF7L2* has the same spliced exons in humans and in mice, thus we considered that the same splicing machinery for *TCF7L2* should be present. We hypothesized that the C construct would produce mRNAs containing an additional spliced exon corresponding to the introduction of the SNP region while the T construct would skip this exon. 72 h after transfection, RNA was extracted and RT-PCR was performed with primers specific to human exons 4 and 5. The PCR products were tested, isolated, and sequenced. No amplification was observed in control cells and cells transfected with either the C/C or T/T construct showed similar amplification bands at approximately 160 bp (Figure 1D). The sequence of both bands corresponded to the sequence of exon4-exon5 (166 bp), indicating that the mRNA from our constructs was correctly spliced (intron 4 was absent) but our hypothetical extra exon was not present in cells transfected with the C variant.

## Discussion

Human *TCF7L2* mRNA is alternatively spliced in several tissues, including pancreatic islets of Langerhans (Prokunina-Olsson et al., 2009; Mondal et al., 2010). Certain variants are tissue-specific, such as variants containing exon 16 which so far have only been found in islets or colon (Prokunina-Olsson et al., 2009). Mouse *Tcf7l2* mRNA also has an alternative splicing pattern which is tissue-specific (Weise et al., 2010). However, splicing in mouse beta-cells or isolated mouse islets has never been determined. We show here that not only is mouse *Tcf7l2* mRNA alternatively spliced in MIN6 and isolated mouse islets but that the splicing pattern is similar to that in isolated human islets. In humans islets, Mondal et al. showed that the major variant was 12–17 (exon 12 spliced into exon 17), and other variants were 12-13-17, 12-15-17, 12-15-16-17 (Mondal et al., 2010). In accordance, we found that the major transcript is 12–17, followed by 12-15-17 and 12-13-17. Interestingly, we also detected the presence of variant 12-15-16-17, which in mouse has only been detected in brain and gut (Weise et al., 2010). In humans, exon 16 was only detected in islets, mainly the 12-15-16-17 variant (Mondal et al., 2010), indicating that this conserved isoform might be relevant in islet function.

When analyzing the sequences of the two SNP variants (C/T), we found that they exhibited differential recognition by splicing factors. The C variant could promote splicing and create a new exon inside intron 4, while the T variant could skip this exon. Several studies have investigated the alternative splicing of *TCF7L2* but this extra exon has not yet been identified. We hypothesized that the reason for this lack of detection is that the inclusion of this new exon in the C variant could lead to an mRNA with a premature stop codon. This mRNA would undergo NMD and consequently reduce mRNA levels. NMD is a process that is difficult to detect, as the mRNA is highly unstable and very rapidly degraded. The T variant could result in skipping of this exon, reducing NMD, and finally increasing *TCF7L2* mRNA levels, as others have previously reported (Lyssenko et al., 2007; Pang et al., 2011).

Intron 4 of human *TCF7L2* is unusually long; therefore we created two mini-genes of this intron with either the C/C or T/T genotype. Since the SNP rs7903146 is thought to be associated to altered insulin processing and secretion we chose to investigate its effect in beta-cells. We opted for MIN6 cells for two reasons: firstly, as shown here, they splice *Tcf7l2* mRNA similarly to human *TCF7L2* and secondly they do not have the corresponding genomic region for human rs7903146 in the *Tcf7l2* gene. Transfecting our constructs into MIN6 cells showed that, indeed, exon 4 spliced correctly into exon 5, but the existence of an extra exon within intron 4 was not detected. However, it is important to consider that, by excluding the majority of the intron (78,000 bp), we might have eliminated important information, and the existence of an extra exon could still be likely. Another approach will be to inhibit NMD in human beta-cells, to better address the existence of this exon.

## Abbreviations

ESE: Exon splicing enhancer
ESS: Exon splicing silencer
NMD: Nonsense-mediated decay
PC1: Proprotein Convertase 1
SNP: Single nucleotide polymorphism
T2D: Type 2 Diabetes
TCF7L2: Transcription factor 7-like 2.
